# In vitro hepatic biotransformation of the algal toxin pectenotoxin-2

**DOI:** 10.1016/j.toxcx.2020.100031

**Published:** 2020-03-23

**Authors:** Morten Sandvik, Christopher O. Miles, Alistair L. Wilkins, Christiane Fæste

**Affiliations:** a Norwegian Veterinary Institute, P. O. Box 750 Sentrum, NO-0106, Oslo, Norway; b Biotoxin Metrology, Measurement Science and Standards, National Research Council, 1411 Oxford Street, Halifax, NS, B3H 3Z1, Canada; c Waikato University, Private Bag 3105, Hamilton, 3240, New Zealand

## Abstract

We have investigated the *in vitro* metabolism of pectenotoxin-2 (PTX-2) using primary hepatocytes from Wistar rats in suspension. Purified PTX-2 was rapidly metabolized. Two major and several minor oxidized PTX-2 metabolites were formed, none of which had retention times corresponding to PTX-1, -11, or −13. Hydrolysis products, such as PTX-2 seco acid, were not observed. Preliminary multi-stage LC-MS analyses indicated that the major hepatic PTX-2 metabolites resulted from the insertion of an oxygen atom at the positions C-19 to C-24, or at C-44. The rapid oxidative metabolism may explain the low oral toxicity of PTXs observed in vivo studies.

## Highlights

• PTX-2 is rapidly metabolized in rat hepatocytes.
• Two major and several minor oxidized PTX-2 metabolites were formed.
• The results may explain the low oral toxicity of PTXs observed in *vivo* studies*.*

## 1 Introduction

Pectenotoxins (PTXs) are a group of marine polyether-lactone toxins exclusively produced by dinoﬂagellate algae of *Dinophysis* species (Yasumoto, 1989, Lee et al., 1989, Farabegoli et al., 2018). Long-term observation of their abundance in Northern seas have shown a negative correlation with increasing sea surface temperatures and wind speed, and consequently on the production of PTXs (Hinder et al., 2012). The toxins can get into the food chain in bivalve molluscs and crustaceans that feed on PTXs-containing microalgae. They have been detected shellfish in Australia, Japan, New Zealand and different European countries (Suzuki et al., 2001a, Suzuki et al., 2005, Suzuki et al., 2001b, Miles et al., 2004a). In an assessment on the occurrence of PTX-2 in different shellfish in 2009, the European Food Safety Authority (EFSA) determined the toxin in only 18% of the surveyed shellfish samples, mostly in mussels and oysters (Alexander et al., 2009). Evaluating the risk for human consumers of shellfish, EFSA established an acute reference dose (ARfD) of 0.8 μg PTX-2 equivalents/kg body weight (bw) based on a lowest-observed-adverse-effect-level (LOAEL) of 250 μg/kg bw for intestinal toxicity of PTX-2 observed in mice. Considering the lack of sufficient data, a provisional toxicity equivalent factor (TEF) of 1 was used for PTX-1, PTX-2, PTX-3, PTX-4, PTX-6 and PTX-11, whereas the much less toxic PTX-7, PTX-8, PTX-9, PTX-2 seco acid (SA) and 7-epi-PTX-2 SA were not assigned TEFs (Alexander et al., 2009).

At least 14 PTX analogues have been described, of which PTX-2, PTX-11, PTX-12, PTX-13 and PTX-14 are produced by *Dinophysis*, whereas all others are products of metabolism reactions in shellfish or spiroketal stereoisomers (Sasaki et al., 1998, Suzuki et al., 2003, Miles et al., 2006a; Suzuki et al., 2006; Alfonso et al., 2014). PTX-2 appears to be the main precursor, of which derivatives such as PTX-1, PTX-3, and PTX-6 are formed through progressive oxidative biotransformation in the gut of bivalves (Suzuki et al., 1998, Burgess and Shaw, 2001), although PTX-1 has also been reported in algae (Krock et al., 2008). PTX-2 SA, a hydrolysis product of PTX-2, has been detected in different shellfish species (Daiguji et al., 1998, Suzuki et al., 2001a, Suzuki et al., 2001b, Miles et al., 2004a). Incubation of PTX-2 with homogenates of green-lipped mussels (*Perna canaliculus*) and scallops (*Pecten novaezelandiae*) has revealed that the hydrolysis to PTX-2 SA is enzyme-catalysed (Suzuki et al., 2001a, Suzuki et al., 2001b), and studies with blue (*Mytilus edulis*) and green-lipped mussels have shown that the hydrolytic reaction is rapid and that the enzymes involved are water-soluble (Miles et al., 2004a).

There is a large difference in toxicity between the intraperitoneal (i.p.) and per oral (p.o.) application of PTX-2 to mice (Alexander et al., 2009). Both, incomplete absorption and a high elimination rate due to extensive biotransformation might cause low systemic concentrations after oral application (Masimirembwa et al., 2003). However, little is known about PTX toxicokinetics besides hydrolysis of PTX-2 to PTX-2 SA and oxidation to PTX-1, PTX-3 and PTX-6 in scallops (Suzuki et al., 1998, Burgess and Shaw, 2001). Preliminary data from studies in mice with single oral doses of 0.3, 1 and 5 mg/kg bw PTX-2 suggested low uptake from the gastrointestinal tract and considerable metabolic elimination (Burgess and Shaw, 2001, Alexander et al., 2009). *In vivo* biotransformation processes such as the rapid hydrolysis to non-toxic seco acid forms like PTX-2 SA or the formation of oxidized PTXs like PTX-11 could have contributed to decrease the amount of PTX-2 that was detectable in different organs and blood (MacKenzie et al., 2005).

In mouse bioassays for the determination of toxic potentials, PTX-11 displayed a similar toxicity profile as PTX-2, with an LD_50_ of 0.24 mg/kg bw i. p., but no effects were detected after the application of as much as 5 mg/kg bw p. o. (Suzuki et al., 2006). Since PTX-11is, in contrast to PTX-2, relatively resistant to enzymatic hydrolysis in mussels (MacKenzie et al., 2005), this pathway is probably not mainly responsible for the lack of oral toxicity of the PTXs. The discrepancy regarding the considerable cellular and i. p. toxicity of the PTXs on the one hand, and the lack of toxicity after p. o. administration on the other, could therefore originate from the toxicokinetic characteristics of this compound class. However, toxicokinetic parameters after oral and intravenous application of important PTXs have not been determined or predicted for animals or humans.

Primary hepatocytes or hepatic microsomes represent well-established systems to study drug metabolism *in vitro* (Iwatsubo et al., 1997, Ito et al., 1998, Nagilla et al., 2006, Fagerholm, 2007, Houston and Galetin, 2008). Freshly isolated hepatocytes in suspension have been used in short-term incubations under kinetic conditions to predict *in vivo* clearances. Assays with test substance in excess are used to identify major metabolites. Primary hepatocytes retain the capacity for biotransformation of xenobiotics by different oxidative phase I and conjugative phase II pathways (Blanchard et al., 2006). In the present study, we report the oxidative metabolism of PTX-2 by fresh rat hepatocytes, and the partial characterization of the metabolites by LC-MS/MS analysis.

## 2 Materials and methods

### 2.1 Materials

PTX-2 was purified from an algal bloom in Sognefjorden, Norway (Miles et al., 2004a, Miles et al., 2004b). After purification by application to basic alumina, the fraction containing PTXs was applied to a column of Lichroprep RP-18 (17 × 2 cm; 40–63 μm, Merck, Germany), and the column was eluted by a step-wise gradient of methanol (MeOH) in water. Fractions containing most of the PTX-2 were applied to a 32 × 1 cm Lichroprep RP-18 column. Fractions were eluted by a linear gradient of MeOH in water, and collected and monitored by LC-MS. PTX-1 was a gift from T. Yasumoto (Japan Food Research Laboratories). Hepes buffer and collagenase (type IV) were obtained from Sigma (Buchs, Switzerland). All other chemicals were of analytical grade.

### 2.2 Preparation of primary rat hepatocytes

Isolated rat liver cells were prepared by a two-step collagenase *ex vivo* perfusion from male Wistar rats, weighing 250–300 g and kept under routine laboratory conditions with free access to standard laboratory pelleted feed and water (Seglen, 1976). The hepatocytes were separated by gradient centrifugation (Fæste and Moldes-Anaya, 2016), suspended in PBS, and stored for up to 1 h on ice before use (Sidelmann et al., 1996, Gebhardt et al., 2003). Cell viability was determined microscopically (Leica DMIL microscope; Leica Microsystems GmBH, Wetzlar, Germany), using Trypan blue staining and a Neubauer-counting chamber. About 2.5 × 10^8^ living cells per liver were obtained on average, and only preparations with viabilities >92% were used in the metabolism experiments.

### 2.3 *In vitro* hepatocyte assays under kinetic conditions

The hepatocyte suspension was diluted with KRB–HEPES buffer pH 7.4 (Krebs-Ringer buffer containing 145 mM NaCl, 5.4 mM KCl, 0.8 mM MgSO_4_, 0.33 mM Na_2_HPO_4_, 0.34 mM KH_2_PO_4_, 2 mM CaCl_2_, and 20 mM HEPES) to a final assay concentration of 2.5 × 10^6^ cells/mL. The incubations were performed in capped round-bottom glass tubes in a total assay volume of 5 mL. After pre-incubation for 5 min at 37 °C in a shaking water bath, 5–25 μL of a PTX-2 stock solution containing 1 mg/mL PTX-2 in MeOH were added, resulting in initial concentrations of 1–5 μg/mL PTX-2 in the assay. Aliquots (300 μL) were taken after 0, 2.5, 5, 7.5, 10, 15, 30, and 60 min of incubation and directly transferred into 300 μL ice-cold acetonitrile (ACN) in Eppendorf tubes. Two control samples were included in the experiments, a blank matrix control and a PTX-2 stability control. After centrifuging all samples at 16,000 *g* for 1 min (5415D centrifuge; Eppendorf AG, Hamburg, Germany), the supernatants were transferred to HPLC vials for analysis by liquid chromatography mass spectrometry (LC-MS) to determine the half-life of PTX-2 in rat hepatocytes.

### 2.4 *In vitro* hepatocyte assays with substrate saturation

Six replicate preparative incubations each containing 160 μg PTX-2 were performed for 30 min as described above. The reactions were stopped by transferring the complete 5 mL-assays into 10 mL ice-cold ACN in 50 mL glass tubes and centrifuged at 9000 *g* for 10 min (J2-MC; Beckman Instruments, Palo Alto, CA, USA). The six supernatants were combined, and evaporated to dryness *in vacuo* using a rotary evaporator (Büchi R-200, Flavil, Switzerland). The residue was dissolved in 50 mL water and subsequently re-extracted with diethyl ether (3 × 30 mL). The combined ether extracts were evaporated to dryness *in vacuo*, re-dissolved in 1 mL ACN–water (40–60) and submitted to preparative high performance liquid chromatography (HPLC).

### 2.5 Separation of PTX-2 metabolites by preparative HPLC

The concentrated sample was fractionated by flash column chromatography using a glass column (10 × 130 mm), slurry-packed with 5 g LiChroprep RP-18 (40–63 μm; Merck, Germany) in ACN and conditioned with 40% ACN in water. The column was eluted using a step-wise gradient of 40%, 60%, and 80% ACN in water (5 mL per step) and, finally, 20 mL 100% ACN. Thirty-five 1-mL fractions were collected manually into 10 mL glass tubes. Substance concentrations in the fractions were determined by LC-MS, and accordingly, the fractions 4, 5, 6, 7, 8 and 12 were combined for further analysis. They were concentrated to 300 μL by evaporation in a nitrogen stream at 40 °C and in the following purified by preparative HPLC. Preparative HPLC was performed on a 250 × 10 mm, 5 μm, Supelcosil LC-18 DB semi-preparative column (Supelco, Bellefonte, PA, USA) using a Gilson model 321 pump, a model 232 XL sampling injector and a 206 fraction collector (Gilson, Middelton WI, USA). Metabolites were eluted with isocratic mixtures of ACN and water at 3 mL/min. Fractions were collected manually by monitoring absorbance at 235 nm with a model 1100 series G1315A diode array detector (Agilent, Santa Clara, CA).

### 2.6 LC-MS and LC-MS^n^ analysis

LC-MS analyses of centrifuged aliquots from the kinetic assays were performed under reversed phase conditions using a Symmetry C_18_ column (5 μm, 3.9 × 150 mm) (Waters, Milford, MA, USA) and a binary LC pump (Finnigan Mod. 250) coupled to an Autosampler Plus (Thermo Electron Corporation, San Jose, CA, USA). 5–20 μL samples were injected and eluted using a linear gradient starting with ACN/water (40:60, v/v, both containing 0.1% formic acid and 2 mM ammonium formate) and rising to 100% ACN in 20 min. Isocratic elution with 100% ACN was maintained for 5 min before the eluent was returned to the starting conditions. The flow rate was set to 1 mL/min.

Separated metabolite fractions from the preparative HPLC purification were eluted with a linear gradient from 40% to 100% ACN in 40 min using a Symmetry C_18_ column (5 μm, 4.6 × 250 mm) (Waters, Milford, MA, USA). The HPLC system was coupled by an electrospray ionization (ESI) interface to an LCQ linear ion trap mass spectrometer (Thermo Electron Cooperation). The instrument was operated in MS or MS^n^ full-scan positive ion mode, and spectra were collected from *m*/*z* 350–1200. Typical ESI parameters were a spray voltage of 3.5 kV, desolvation temperature at 250 °C, source temperature at 100 °C and cone gas and desolvation gas at 40 and 600 L/h N_2_, respectively. The mass spectrometer was operated in MS^n^ mode with argon as collision cell gas at 1 × 10^−3^ Torr. Ionization and MS^n^ collision energy settings were 40–45 eV. Total ion chromatograms (TIC) were measured for all incubation aliquots, and the respective peak retentions, MS, MS^2^ and MS3 data of PTX-2 and potential metabolites were obtained.

### 2.7 Calculation of the hepatic clearance of PTX-2 in rats

The *in vitro* assay clearance (CL_assay_) of PTX-2 was calculated from the hepatocyte incubations run with first order kinetics (CL_assay_ = V_assay_ × ln2/*t*_1/2_ [Lh^−1^]) using the assay volume (V_assay_) and the disappearance half-life (*t*_1/2_) of the parent compound as determined from the slope of the linear regression from a semi-logarithmic concentration vs. time plot (Obach, 1999). The intrinsic clearance (CL_int_), which is independent of the experimental conditions (CL_int_ = CL_assay_ x HC x rL/N [L (h x kg)^−1^]) [15] was derived from CL_assay_ under consideration of the number of hepatocytes in the assay (N), the rat hepatocellularity (HC ~ 1.1 × 10^8^/g liver) (Smith et al., 2008), and the rat liver weight (rL ~ 33 g/kg bw) (Lin and Lu, 1998). In this study, we did not consider potential protein binding of PTX-2 in the hepatocyte incubation and did not include the fraction unbound in the assay (fu_assay_ = 1) into the calculations (Obach, 1996). The *in vivo* hepatic blood clearance (CL_b_) was calculated from CL_int_ by applying the non-restricted well-stirred model (CL_b_ = CLint x Q/(CL_int_ + Q) [L (h x kg)^−1^]) (Houston, 1994), considering the hepatic blood flow in rat (Q ~ 4.2 L (h x kg)^−1^) (Lin and Lu, 1998). Potential binding to blood components *in vivo* was not considered in this study and thus the fraction unbound in blood (f_ub_) set to 1. The maximum bioavailability (F_max_) of PTX-2 in rats was estimated (F_max_ = F_a_ x (1 – (CL_b_/Q) under the assumption of complete absorption from the gastrointestinal tract (F_a_ = 1) (De Buck et al., 2007) and negligible non-hepatic elimination. F_max_ was ranked according to the criteria for low (0–10%), moderate (10–30%) and high bioavailability (up to 100%) (Obach, 1996).

## 3 Results and discussion

### 3.1 Identity of isolated PTX-2 by LC/MS and LC/MSMS

**Fig. 1:**
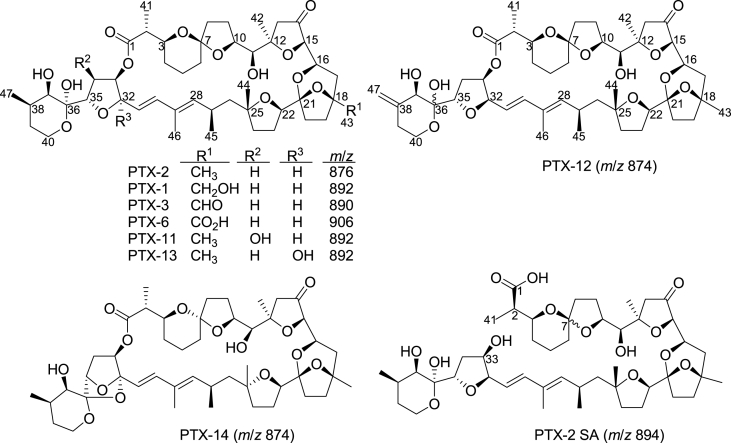
PTXs in algae and shellfish. Functional group patterns and *m*/*z* values of [M + NH_4_]^+^ ions are shown.

PTX-2 (Fig. 1) was purified from an algal bloom in Sognefjorden, Norway (Miles et al., 2004a, Miles et al., 2004b). Reverse-phase chromatography of the ethereal extract, followed by preparative HPLC, provided 13 mg pure PTX-2 with ca. 80% recovery. NMR and LC–MS confirmed the structure and purity of PTX-2 isolated by this method (Miles et al., 2004a). The MS/MS product ion spectrum of PTX-2 was essentially identical to the published PTX-2 reference spectrum, showing the same fragments (Gerssen et al., 2008).

### 3.2 Kinetics of PTX-2 elimination and metabolite formation in the rat hepatocyte assay

**Fig. 2:**
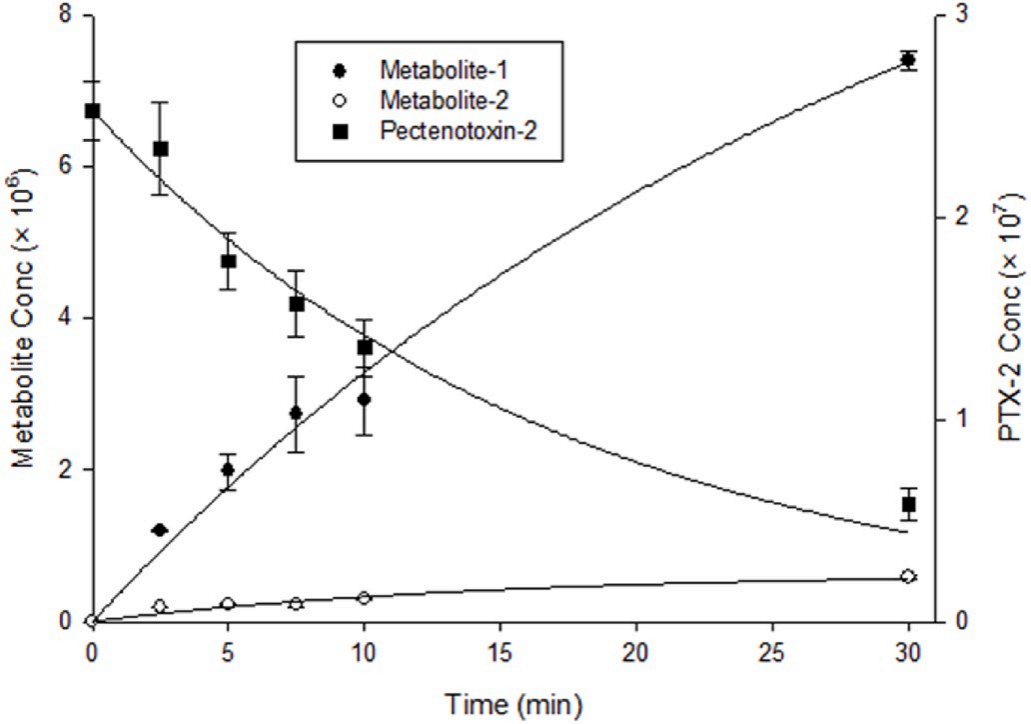
Concentration-versus-time curves of PTX-2 (*m/z* 881) (▪), M1 (*m/z* 897) (•) and M2 (*m/z* 897) (◦), determined by LC–MS during incubation of PTX-2 (1.4 μg/mL) in primary rat hepatocytes. Regression lines are best fits for first-order kinetics.

PTX-2 was rapidly metabolized in freshly isolated rat hepatocytes. The disappearance of the parent compound and the formation of the two potential main metabolites M1 and M2 are plotted as a function of the incubation time in Fig. 2. The assay half-live of PTX-2 was comparable in incubations with different start concentrations, indicating linear kinetics (Table 1). At all concentrations, PTX-2 underwent rapid metabolic transformation with 17–20% remaining unchanged after 30 min of incubation. Metabolism of PTX-2 to other PTX derivatives by shellfish, and biosynthesis of analogues in algae, has provided a growing library of compounds for comparison (Yasumoto et al., 1985, Suzuki et al., 1998, Suzuki et al., 2006, Daiguji et al., 1998, Miles et al., 2004b, Miles et al., 2006b, Halim and Brimble, 2006).

**Table 1:** PTX-2 half-lives in primary rat hepatocytes and predicted toxicokinetic parameters.

PTX-2 (μg/mL)	t_½,assay_ [h]	CL_int_ [l/(h·kg)]	CL_b_ [l/(h·kg)]	f_max_ [%]
1.4	0.19	8.74	2.84	32
2.6	0.16	10.44	3.00	29
4.8	0.20	8.58	2.82	33

### 3.3 Prediction of the *in vivo* hepatic clearance and maximum bioavailability of PTX-2 in rats

The intrinsic clearance (CL_int_) describes the efficiency of the metabolism enzymes for a compound. CL_int_ can be scaled to the *in vivo* blood clearance (CL_b_), which is a measure for the systemic elimination, by considering physiological parameters such as hepatocellularity, liver weight, a model of hepatic extraction such as the well-stirred model, and the hepatic blood flow. Assuming a negligible influence of protein binding and unrestricted absorption, the *in vitro*-based estimate of *in vivo* data predicted a moderate hepatic blood clearance and bioavailability for PTX-2 in rats. After oral uptake, up to 32% of the compound is predicted to remain unmetabolized after the first pass through the liver (Table 1). However, the few available *in vivo* results from mouse studies with oral PTX-2 application indicted a rather limited gastrointestinal absorption (Burgess and Shaw, 2001, Alexander et al., 2009), so that the actual bioavailability is probably considerably lower than the predicted maximal f_max_ value. Furthermore, caution should be exercised for compounds undergoing extensive metabolism due to the potential for significant extrahepatic metabolism (Obach, 1996, Soars et al., 2002). If PTX-2 was additionally cleared by mechanisms other than liver metabolism (e.g. extrahepatically by the kidneys, plasma enzymes, protein binding or chemical instability), the total clearance would be the sum of the individual clearances, including the hepatic blood clearance.

### 3.4 Metabolite profile after incubation of PTX-2 with rat hepatocytes

**Fig. 3:**
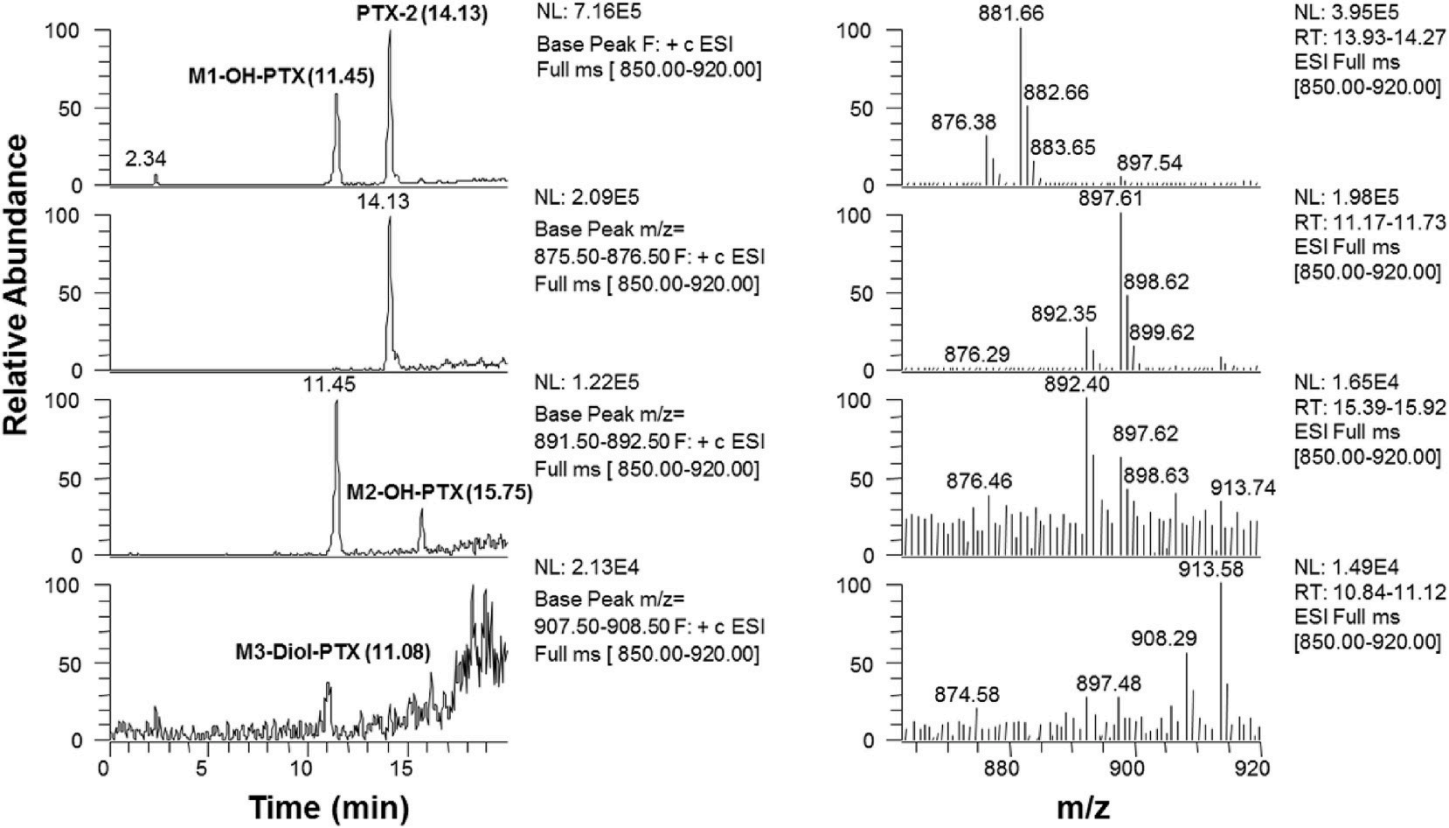
LC-MS chromatograms (scan mode *m/z* 850–920, [M + NH_4_]^+^, conditions of LC-MS^n^ analysis) showing PTX-2 and three potential metabolites in 30 min-incubations with rat hepatocytes (left panel). LC-MS spectra displaying [M + NH_4_]^+^ and [M+Na]^+^ ions of PTX-2 and the PTX-2 metabolites (right panel).

LC-MS analysis of 30 min-incubations with PTX-2 in primary rat hepatocytes revealed the presence of a major monohydroxylated peak (M1) eluting at 11.45 min, which showed [M + NH_4_]^+^ and [M+Na]^+^ at *m/z* 892 and 897, respectively (Fig. 3). A second, minor monohydroxylated peak (M2), or more likely an epoxide, eluted at 15.75 min and thus later than PTX-2 at 14.13 min. It also produced [M + NH_4_]^+^ and [M+Na]^+^ with *m/z* 892 and 897, respectively. Moreover, a minor dihydroxylated product (M3), eluting at 11.08 min, showed [M + NH_4_]^+^ and [M+Na]^+^ at *m*/*z* 908 and 913, respectively.

### 3.5 LC/MS and LC/MS^n^ analyses of the isolated metabolites

**Fig. 4:**
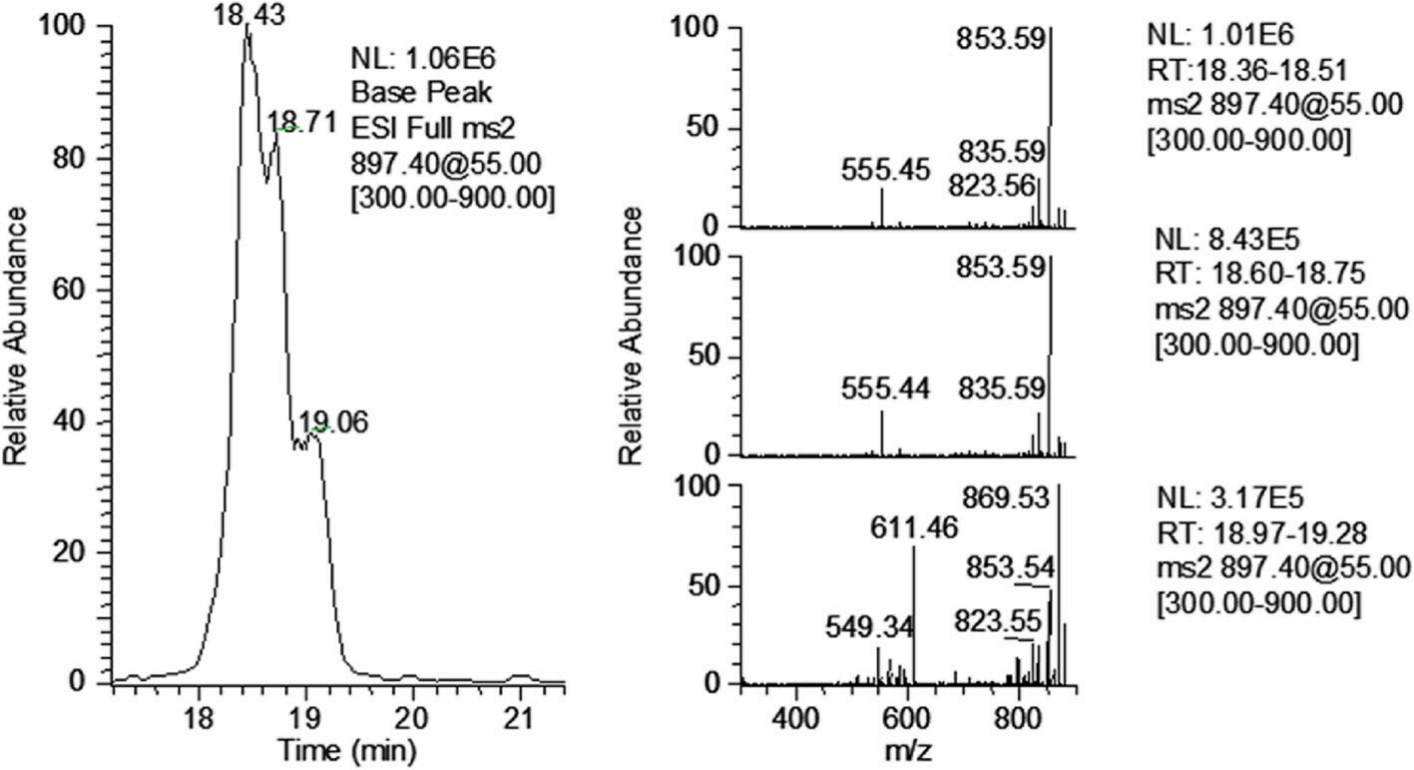
LC-MS^2^ chromatogram of monohydroxylated M1 indicating the presence of three isomeric OH-PTX-2 species with [M+Na]^+^ ions at *m/z* 897 (left panel). The species with retention times 18.43 and 18.71 min produce a significant fragment ion at *m*/*z* 555 for [M+Na]^+^ in MS^2^ spectra, whereas the third species with retention time 19.06 min produces a significant fragment ion at *m*/*z* 611 (right panel).

When combined 30-min incubations were fractionated and subsequently subjected to preparative HPLC using a longer, 250 mm-column and a slower ACN–water gradient, the fraction containing the major monohydroxylated M1 peak was resolved into three partially overlapping peaks (Fig. 4). Analysis of MS^2^ spectra ([M+Na]^+^) suggested that the fraction of monohydroxylated M1 included contributions from different OH-PTX-2 species. The two signals with retention times 18.43 and 18.71 min (leading edge of the main peak) produced both a *m/z* 555 fragment ion, and the minor signal at 19.06 min, possibly consisting of isomeric compounds (tailing edge of the peak) produced a significant fragment ion with *m/z* 611.

### 3.6 Characterization of the potential hepatic PTX-2 metabolites

**Fig. 5:**
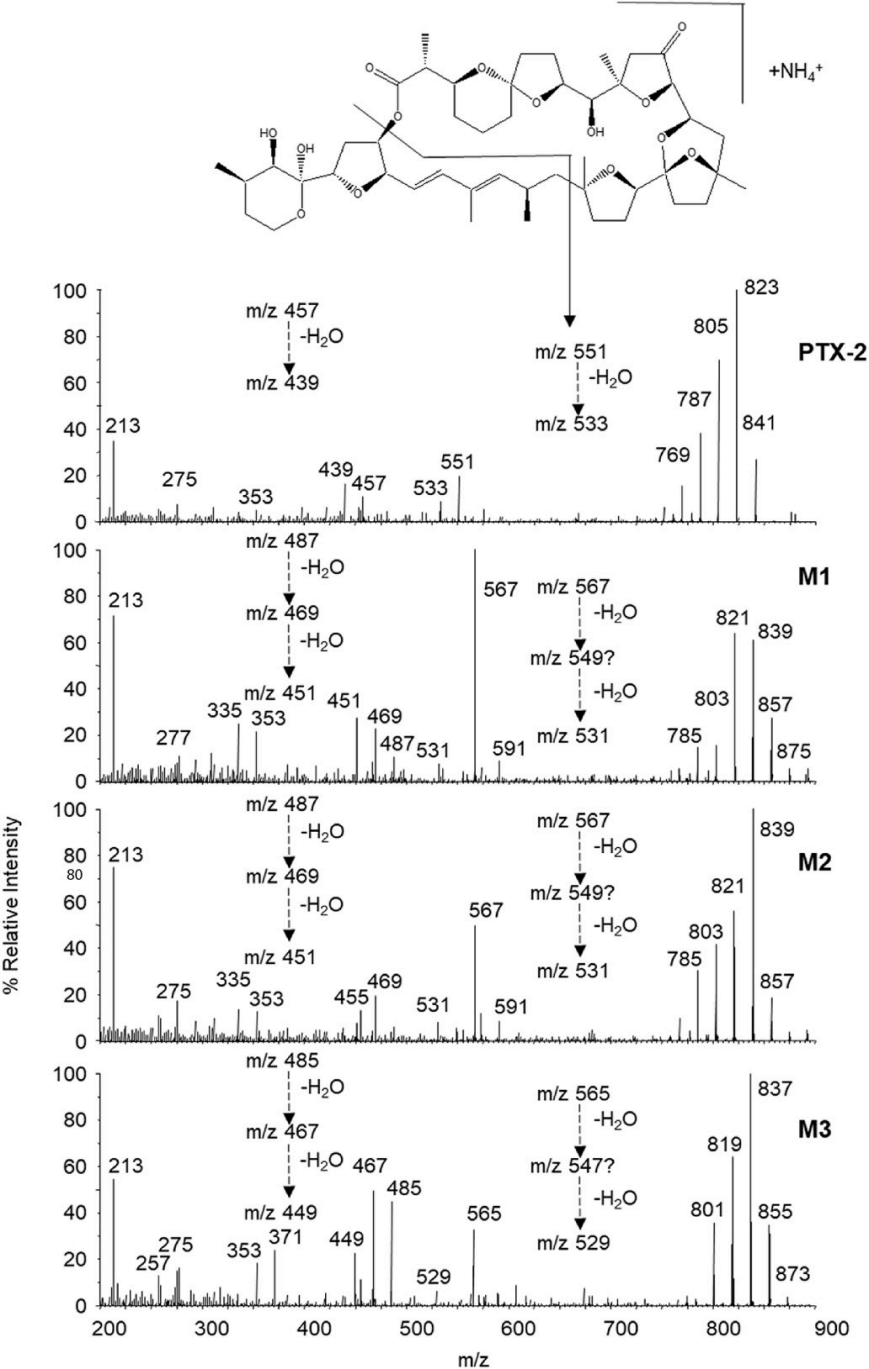
Proposed fragmentations observed in positive MS/MS spectra of [M + NH_4_]^+^ ions from PTX-2 and the PTX-2 metabolites M1, M2 and M3 with comparison of the fragmentation pathways.

The [M + NH_4_]^+^ MS^2^ and MS^3^ spectra of the monohydroxylated metabolites M1 and M2 showed sequential losses of water (−18 Da) resulting in the fragment ions of *m/z* 857 → 839 → 821 → 803 → 785 → 767, and M3 with fragment ions of *m/z* 873 → 855 → 837 → 819 → 801, comparable to the distinct fragmentation pattern of PTX-2 (*m/z* 869 → 851 → 823 → 805 → 787 → 769) (Fig. 5). Moreover, we observed the combined loss of CO_2_ (−44 Da) and a 298 Da-fragment (in total −342 Da) from the molecule ions, producing the typical fragment ions at *m/z* 549 (M1, M2) and *m/z* 533 (PTX-2), that again can lose water. Analogous typical fragments were found in the MS^2^ spectra of numerous other PTXs, including PTX-2 SA, 7-*epi*-PTX-2 SA, PTX-12a, PTX-12 b and PTX-2 (Miles et al., 2004b, Gerssen et al., 2008). Their presence in the spectra of M1 and M2 demonstrates that the metabolites are not hydroxylated in the upper region of the PTX skeleton, but potentially in the vicinity of R1 (Fig. 1), in the lower right region of the molecule (Fig. 5).

In contrast, the characteristic loss of −342 Da was not observed in the MS^2^ and MS^3^ spectra of the minor monohydroxylated metabolite species observed at a higher retention time (Fig. 4) and the dihydroxylated M3 (Fig. 3). In MS ([M+Na]^+^), both afforded *m*/*z* 611, whereas *m/z* 555 was the typical fragment for the dominant monohydroxylated metabolites. This difference could be attributable to the presence of an added hydroxyl group in the upper region of the PTX skeleton. It appears to be probable that the minor monohydroxylated metabolite showing *m/z* 611 and dihydroxylated M3 are oxygenated at the same position in the upper PTX region. It is also probable that the second site of oxygenation in M3 is in the same position in the lower portion of the PTX skeleton as for the dominant monohydroxylated products affording the *m*/*z* 555 ion in [M+Na]^+^ and *m/z* 567 ion in [M + NH_4_]^+^ MS and MS^2^ spectra.

The [M + NH_4_]^+^ MS spectra of the monohydroxylated M1 and M2 (Fig. 3) were distinctly different to those of known PTX derivatives with the same molecular weights including PTX-2 (Yasumoto et al., 1984, Miles et al., 2004b), PTX-11 (Suzuki et al., 2006), and PTX-13 (Miles et al., 2006a). Furthermore, the retention times of the potential PTX-2 biotransformation products M1, M2 and M3 were different from that of an authentic PTX-1 standard, although PTX-1 has the same molecular formula (Krock et al., 2008). It can thus be assumed that the possible metabolic hydroxylation sites in the PTX-2 molecule, which we roughly localized by LC-MS^n^ analysis, do not correspond with the sites that are known to be oxygenated during the biosynthesis of PTX-1, PTX-11, and PTX-13. Formal structure elucidation of the hepatic metabolites detected in the present study awaits the availability and purification of a quantity of each compounds that is sufficient for detailed NMR analyses.

## CRediT authorship contribution statement

**Morten Sandvik:** Conceptualization, Methodology, Data curation, Writing - original draft, Visualization, Investigation. **Christopher O. Miles:** Data curation, Writing - original draft, Writing - review & editing, Investigation. **Alistair L. Wilkins:** Visualization, Writing - review & editing. **Christiane Fæste:** Investigation, Writing - review & editing.

## Declaration of competing interest

The authors declare that they have no known competing financial interests or personal relationships that could have appeared to influence the work reported in this paper.
